# Advances in the study of acetaminophen-induced liver injury

**DOI:** 10.3389/fphar.2023.1239395

**Published:** 2023-08-04

**Authors:** Xinghui Li, Jiaqi Ni, Li Chen

**Affiliations:** ^1^ West China School of Pharmacy, Sichuan University, Chengdu, China; ^2^ Department of Pharmacy, Evidence-Based Pharmacy Center, West China Second University Hospital, Sichuan University, Chengdu, China; ^3^ Key Laboratory of Birth Defects and Related Diseases of Women and Children, Sichuan University, Ministry of Education, Chengdu, China

**Keywords:** drug-induced liver injury, acetaminophen-induced liver injury, diagnosis, screening, prevention and management

## Abstract

Acetaminophen (APAP) overdose is a significant cause of drug-induced liver injury and acute liver failure. The diagnosis, screening, and management of APAP-induced liver injury (AILI) is challenging because of the complex mechanisms involved. Starting from the current studies on the mechanisms of AILI, this review focuses on novel findings in the field of diagnosis, screening, and management of AILI. It highlights the current issues that need to be addressed. This review is supposed to summarize the recent research progress and make recommendations for future research.

## 1 Introduction

Drug-induced liver injury (DILI) is a term used to describe the unintended damage to the liver that may be caused by commonly used drugs, which is usually an under-recognized or under-diagnosed etiology of liver injury (Devarbhavi et al., 2021). DILI is one of the main reasons for the termination of drug development, post-marketing warnings or restrictions, or post-approval withdrawal. The incidence of DILI is estimated to be 14–19 cases per 100,000 people globally. Although drug-induced acute liver injury (ALI) is uncommon, accounting for less than 1% of ALI (Sgro et al., 2002), hepatotoxicity of drugs is the most common cause of acute liver failure (ALF) in countries such as Europe, the United States, and Japan (Hillman et al., 2016). The liver is the central site of the biotransformation (metabolism) of drugs entering the body, where drugs undergo different degrees of structural changes with the action of metabolic enzymes. Thus, the liver is vulnerable to the adverse effects of many chemical compounds, dietary supplements, and herbs (Garcia-Cortes et al., 2020). Therefore, it is necessary to propose practical methods for diagnosing, screening, preventing, and managing DILI, which significantly improves clinical outcomes, patient health, and the research and development of novel drugs.

Based on the mechanism, DILI is divided into intrinsic and idiosyncratic types. The intrinsic type is usually caused by the direct toxicity of drugs (Andrade et al., 2019a). Intrinsic liver injury is generally dose-dependent and predictable, with onset within hours to days after the drug exposure. In contrast, idiosyncratic liver injury (IDILI) occurs mainly in patients susceptible to specific medications. It is usually not dose-dependent and without association with the drug regimen or route of administration (Tujios and Fontana, 2011). IDILI has a long and unpredictable latency period, which is also influenced by multiple factors, including drugs, hosts, and environment (Andrade et al., 2019a). Currently, the incidence of IDILI is low, and its mechanisms are complicated. Moreover, the DILI identified during drug development is mainly intrinsic rather than idiosyncratic. Therefore, this review focuses on intrinsic DILI. The most typical drug causing intrinsic DILI in developed countries such as Europe and the United States is acetaminophen (APAP) (Russo et al., 2004). APAP is one of the most commonly used drugs in the household due to its combination of antipyretic and analgesic effects with a dose lower than 4 g per day for healthy adults. APAP-induced liver injury (AILI) is the most common cause of ALF in the United States and Europe, with more than 50% of ALF cases caused by AILI (Andrade et al., 2019b). AILI mainly occurs after unintentional overdose, including a single overdose or several consecutive days at a daily dose of over 4 g. As a result, active monitoring and management of AILI can significantly reduce the incidence of ALF and improve patients’ prognoses.

The mechanism of AILI is complicated, involving various signaling pathways. Moreover, many factors, such as alcohol abuse, nutrition status, underlying diseases, and concomitant medication use, may lower the toxicity threshold of APAP, making AILI less predictable (Andrade et al., 2019a). This review summarizes the current generally accepted mechanisms of AILI. It discusses some novel signaling pathways under investigation to indicate potential targets for therapeutic interventions and new drug development. The diagnosis of AILI relies on a high degree of suspicion and careful exclusion of other possible etiologies. However, this approach relies on the subjective description of the patients or their families and the physician’s knowledge of the disease, making the diagnosis of AILI challenging. Therefore, diagnostic and screening methods that can specifically identify AILI or preemptively indicate the risk of AILI during drug treatment are needed. Specific biomarkers may be adopted to predict AILI as they usually change significantly during the early stage of liver injury. This review discusses some popular biomarkers currently under investigation and highlights promising areas for future research. Due to the lack of *in vitro* models capable of accurately screening for hepatotoxic drugs, DILI is one of the leading causes of clinical trial failures and post-approval withdrawal. This review describes some *in vitro* models currently under investigation. The treatment option for AILI is limited, focusing mainly on APAP-induced mitochondrial oxidative stress. N-acetylcysteine (NAC) is the only drug approved by the U.S. Food and Drug Administration (FDA) to treat APAP overdose (Chowdhury et al., 2020). Although most patients will recover completely with NAC treatment, some may develop chronic DILI and even ALF in severe cases, eventually leading to liver transplantation. These patients are exposed to extremely high doses of APAP, or their liver injuries have progressed to severe status before seeking medical help. Consequently, there is a demand for more prevention and treatment options superior to NAC regarding efficacy and safety. This review exemplifies potential targets and signaling pathways that may be used for new drug development for AILI. The application of nanoparticle-mediated drug delivery that can improve the efficacy of NAC is proposed. Some natural products with possible prevention and treatment capabilities for AILI are discussed.

**FIGURE 1:**
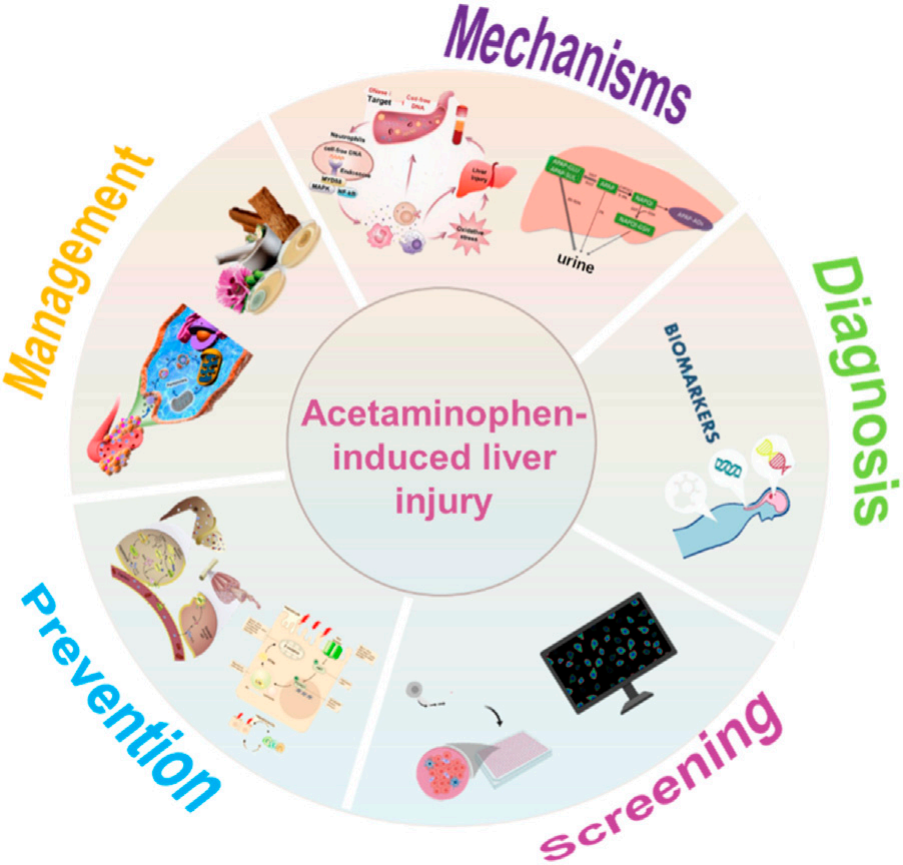
Structure of the entire review.

This review starts with the general description and classification of DILI, further summarizes the underlying mechanisms of AILI, and highlights the current research and novel findings in the diagnosis, screening, and management of AILI. The current challenges and potential future research are discussed. Figure 1 illustrates the structure of the entire review, including sections on the mechanisms, diagnosis, screening, prevention, and management of AILI.

## 2 Mechanisms of AILI

### 2.1 Classification of DILI

DILI can be classified into hepatocellular, cholestatic, and mixed liver injury (with features of both) based on its clinical manifestations and pathological features (Zimmerman, 2000). Hepatocellular liver injury is the most prevalent and easily identified type, accounting for approximately 90% of DILI. The main features of hepatocellular liver injury include hepatocyte necrosis and infiltration of intrahepatic lymphocytes and eosinophils, often accompanied by mild cholestasis, inflammatory reaction, significant elevation of serum aspartate aminotransferase (AST) and alanine aminotransferase (ALT) levels, and moderate elevation of gamma-glutamyl transpeptidase (GGT) and alkaline phosphatase (ALP) levels (Larrey, 2000). Cholestatic liver injury is characterized histologically by cholestasis in the capillary bile ducts. It is often accompanied by jaundice, bile duct hyperplasia or injury, portal phlebitis, significantly elevated GGT and ALP levels, and slightly elevated AST and ALT levels. Mixed liver injury has all these characteristics, with an overall increase in ALP and serum ALT/AST ratio (ALT/AST). The R-score is used clinically as a reference to determine the phenotype of DILI and is defined as

$$R-score=\frac{\left[ALT\right]/{ALT}_{UNL}}{\left[ALP\right]/{\left[ALP\right]}_{UNL}}.$$

Where [ALT] and [ALP] are the measured values of serum ALT and ALP levels of patients, [ALT]_UNL_ and [ALP]_UNL_ are the average upper normal limits. R > 5 indicates mainly hepatocellular liver injury; R < 2 indicates mainly cholestatic liver injury; and 2 < R < 5 indicates mixed liver injury (Quintás et al., 2021). However, R-score cannot be used as a direct criterion to determine the phenotype of DILI because these parameters are not specific to DILI and thus need to be differentiated from other diseases. In addition, minor differences in how R-score is calculated may lead to discrepancies in defining the phenotype of liver injury.

Depending on the disease’s duration, DILI can be divided into acute and chronic types. Acute DILI was mostly recognized with an acute onset, while chronic DILI was widely under-recognized. The American College of Gastroenterology defines chronic DILI as the failure to return to previous bilirubin levels, previous liver enzyme levels, and other signs and symptoms of progressive liver disease (e.g., portal hypertension, hepatic ascites, coagulation abnormalities, and hepatic encephalopathy) within 6–9 months after the onset of DILI (Chalasani et al., 2021). Another study showed that chronic DILI accounts for around 13.6% of all DILI, and about 15%–20% of acute DILI can develop into chronic DILI (Chalasani et al., 2015). Among them, cholestatic DILI is more likely to develop into chronic DILI than hepatocellular DILI.

### 2.2 Potential mechanisms of AILI

AILI is one of the leading causes of ALF in many countries, with 9% of patients suffering from APAP-induced ALF with poor prognoses and requiring liver transplantation (Tujios and Lee, 2018). APAP, one of the typical household medications, is used by more than 10 million people every day for toothache, headache, menstrual pain, arthritis, fever, and other symptoms. If the dose exceeds the recommended daily dose (4 g per day for healthy adults), there is a high possibility for AILI (Larson et al., 2005). Moreover, a therapeutic dose of APAP may also induce AILI in patients with liver diseases, alcoholism, or fasting. Therefore, to detect AILI promptly and control the progression of the disease, it is necessary to understand its mechanism of action.

#### 2.2.1 Acetaminophen metabolism

**FIGURE 2:**
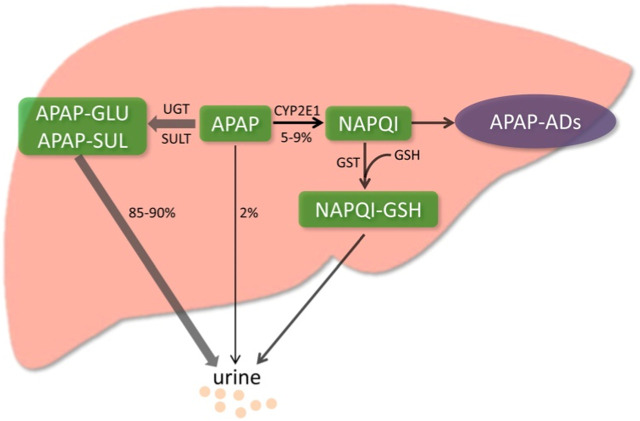
Metabolic activation pathway of acetaminophen. Generally, NAPQI is detoxified by conjugating with GSH. However, excessive NAPQI depletes GSH following APAP overdose, leading to the formation of APAP protein adducts (APAP-Ads) through the covalent binding of sulfhydryl groups in cellular proteins. (Yan et al., 2018; reprinted from Redox Biology with permission).

When APAP is administered at the therapeutic dose, approximately 85%–90% of APAP is metabolized by phase II conjugating enzymes and excreted in the urine (Yan et al., 2018), of which about 50% is converted to APAP-GLU by UDP-glucuronosyltransferase (UGT) and around 30% is converted to APAP-SUL by sulfotransferase (SULT) (Slitt et al., 2003). About 2% of APAP is excreted as a prototype in the urine. Only 5%–9% of APAP is metabolized by cytochrome P450 (CYP) enzymes, mainly by CYP2E1 converted to N-acetyl-p-benzoquinone imine (NAPQI) (Lancaster et al., 2015). NAPQI is an active metabolite detoxified by rapid binding to glutathione (GSH), which is abundant (∼10 mM) in the liver (Chowdhury et al., 2020). The conjugate NAPQI-GSH is first excreted into the bile and then degraded in other organs, such as the kidney, and the degradation products are eventually excreted in the urine (Mcgill and Jaeschke, 2013). However, when APAP overdoses, a significant amount of NAPQI will be produced and deplete the limited storage of GSH in the cytoplasm and mitochondria. The excessive NAPQI will covalently bind to cellular proteins with sulfhydryl groups, especially to mitochondrial proteins (Qiu et al., 1998). The mitochondria and cytoplasm are exposed to reactive oxygen species (ROS), leading to mitochondrial oxidative stress and dysfunction (Figure 2). This process eventually leads to hepatocyte death (Moles et al., 2018). In addition to the recognized oxidative stress and dysfunction of mitochondria, processes such as endoplasmic reticulum stress, microcirculatory dysfunction, hepatocyte regeneration, and sterile inflammatory response have been identified to be involved in the mechanism of AILI (Yan et al., 2018).

#### 2.2.2 Major mechanisms of mitochondrial oxidative stress and dysfunction

The c-Jun N-terminal kinase (JNK) belongs to a subgroup of the mitogen-activated kinases (MAPKs). When NAPQI is depleted of GSH, the excessive NAPQI disrupts the normal antioxidant capacity of mitochondria. This leads to enhanced superoxide production and, ultimately, oxidative/nitrosative stress, which activates JNK (Weston and Davis, 2007). Phosphorylated JNK (p-JNK) translocates to mitochondria and causes mitochondrial electron transport chain (ETC) dysfunction and increased ROS release. The accumulated ROS continues to induce JNK phosphorylation, and the sustained activation of JNK allows for the amplification of ROS, resulting in a cycle of activation (Win et al., 2016). p-JNK induces the opening of the mitochondrial permeability transition (MPT) pore, increasing the mitochondrial permeability and the pore transition (Du et al., 2016). This ultimately leads to DNA breaks and then cell necrosis. However, it is worth noting that the effect of JNK on mitochondria is concentration-dependent. When the ingested dose of APAP was low, JNK exhibited a transient activation state. Thus, the increase in mitochondrial permeability it induced was reversible (Hu et al., 2016).

Nuclear factor erythroid 2-related factor 2 (Nrf2) is a transcription factor encoded by the NFE2L2 gene that NAPQI can indirectly activate. The activated Nrf2 promotes the transcription of antioxidant enzymes, including quinone oxidoreductase 1 (NQO1), heme oxygenase 1 (HO-1), and microsomal epoxide hydrolase, and further promotes GSH synthesis (Itoh et al., 1997). These antioxidant enzymes activated by Nrf2 can play a defense role and detoxify NAPQI. It has been shown that the Nrf2 signaling pathway can be activated by other mechanisms apart from the action of NAPQI (Goldring et al., 2004). Ye et al. (2014) showed that fibroblast growth factor 21 (FGF21), overexpressed due to APAP excess, induces peroxisome proliferator-activated receptor to co-activate protein-1α (PGC-1α) expression, increasing the abundance of Nrf2 in the liver. This is a compensatory mechanism that protects against APAP hepatotoxicity. In addition, protein tyrosine phosphatase 1B (PTP1B) is a negative regulator of tyrosine kinase growth factor signaling. Mobasher et al. (2013) demonstrated that the deficiency of PTP1B in mice resulted in the enhancement of the hepatic Nrf2 system, which protected hepatocytes from APAP-induced cell death.

Once activated, the p53 tumor suppressor protein promotes cell survival and repairs genetic damage in response to various stress, such as cellular stress and DNA damage. However, when DNA damage is severe, or ROS levels are incredibly high, cells are permanently killed by p53-mediated cell death (Kruiswijk et al., 2015). Therefore, the role of p53 in different pathological manifestations can be opposite and complex. When APAP overdoses, p53 is activated by oxidative stress. Interestingly, activated p53 inhibits JNK activation during the NAPQI injury phase, thus exerting a protective effect (Huo et al., 2017), whereas it slows down the process of liver repair during the regenerative phase of hepatocytes (Borude et al., 2018).

In addition, mitochondria take up the cytosolic iron released from the disrupted lysosomes (Kon et al., 2010). This also promotes the increase of mitochondrial permeability and pore conversion induced by oxidative stress, leading to cell necrosis.

#### 2.2.3 Novel pathways being explored on AILI

In addition to the mechanisms mentioned above, some new studies were performed on the mechanisms of AILI. Guo et al. found that mice lacking scavenger receptor A (SRA) were more sensitive to APAP hepatotoxicity. They further investigated the mechanism and found that SRA deletion reduced the anti-inflammatory cytokine IL-10 secretion, exacerbating APAP-induced liver injury. It is because SRA can indirectly promote IL-10 production in damaged hepatocytes and inhibit the activation of the JNK-mediated signaling pathway. This study identifies a hepatoprotective role for the SRA-IL-10 axis. The cMyc gene is one of the critical members of the Myc gene family, which enables unlimited cell proliferation, acquires immortalization functions, and promotes cell division. Kotulkar et al. found that mice specifically knocked out of hepatocyte nuclear factor 4 alpha (HNF4α) exhibited a more substantial degree of liver injury than normal mice after administration of an overdose of APAP, whereas HNF4α-cMyc double knockout mice showed less liver injury with the same dose. Further studies suggested that HNF4α interacted with Nrf2 and promoted GSH replenishment. This study indicates that HNF4α has beneficial effects on liver regeneration and recovery after AILI, a process that cMyc inhibits.

Mas is a G protein-coupled receptor encoded by the oncogene Mas1 that binds explicitly to angiotensin-(1-7) (Hammer et al., 2016; Santos et al., 2018). Recently, studies demonstrated that the renin-angiotensin system (RAS) played a synergistic role with lipid metabolism in the oxidative stress response of hepatocytes (Wu et al., 2016). On this basis, Chen et al. (2023) investigated whether Mas has a beneficial role in AILI. Their experiments showed that Mas1 gene-deficient mice exhibited significant intolerance to APAP under APAP attack, possibly due to insufficient downstream fatty acid oxidation (FAO) and lipophagy. In addition, preadministration of Mas activator AVE0991 resulted in a significant reduction in mitochondrial stress, intrahepatic inflammation, and cell death, suggesting that prophylactic activation of Mas exerts a significant protective effect against APAP overdose by enhancing FAO and lipophagy. Mitsugumin 53 (MG53) has been shown to play a crucial role in membrane repair after cell injury (Jia et al., 2014). Han et al. found that systemic administration of recombinant MG53 (rhMG53) protein in mice would prevent and treat AILI (Han et al., 2022). rhMG53 protein’s hepatoprotective effect was most significant within 1 h of APAP administration, and prophylactic administration of rhMG53 protein significantly improved survival in mice. MG53 interacts directly with mixed lineage kinase domain-like pseudokinase (MLKL) on the plasma membrane, thus improving the diminished MLKL membrane localization and the reduced MLKL polymer synthesis caused by APAP. In contrast, pore formation and oligomerization of MLKL trigger membrane rupture, leading to cell death (Brenner et al., 2013). In summary, this study revealed the protective effects of MG53 on hepatocyte integrity during AILI.

The underlying mechanisms of AILI are complicated, with multiple signaling pathways and multiple phases involved. These intra- and extracellular activities are involved in various aspects of APAP hepatotoxicity, and while they limit cellular stress responses, cause organelle damage, and mediate hepatocyte death, they are also involved in liver repair and regeneration (Yan et al., 2018). Therefore, targeting specific pathways may have beneficial and detrimental effects on patients, which should be thoroughly considered in preclinical studies. In general, multiple pathways of AILI are sources of new drug development, and more studies focusing on the mechanisms of AILI are needed in the future.

## 3 Diagnosis, screening, and prevention of AILI

### 3.1 Novel biomarkers

**FIGURE 3:**
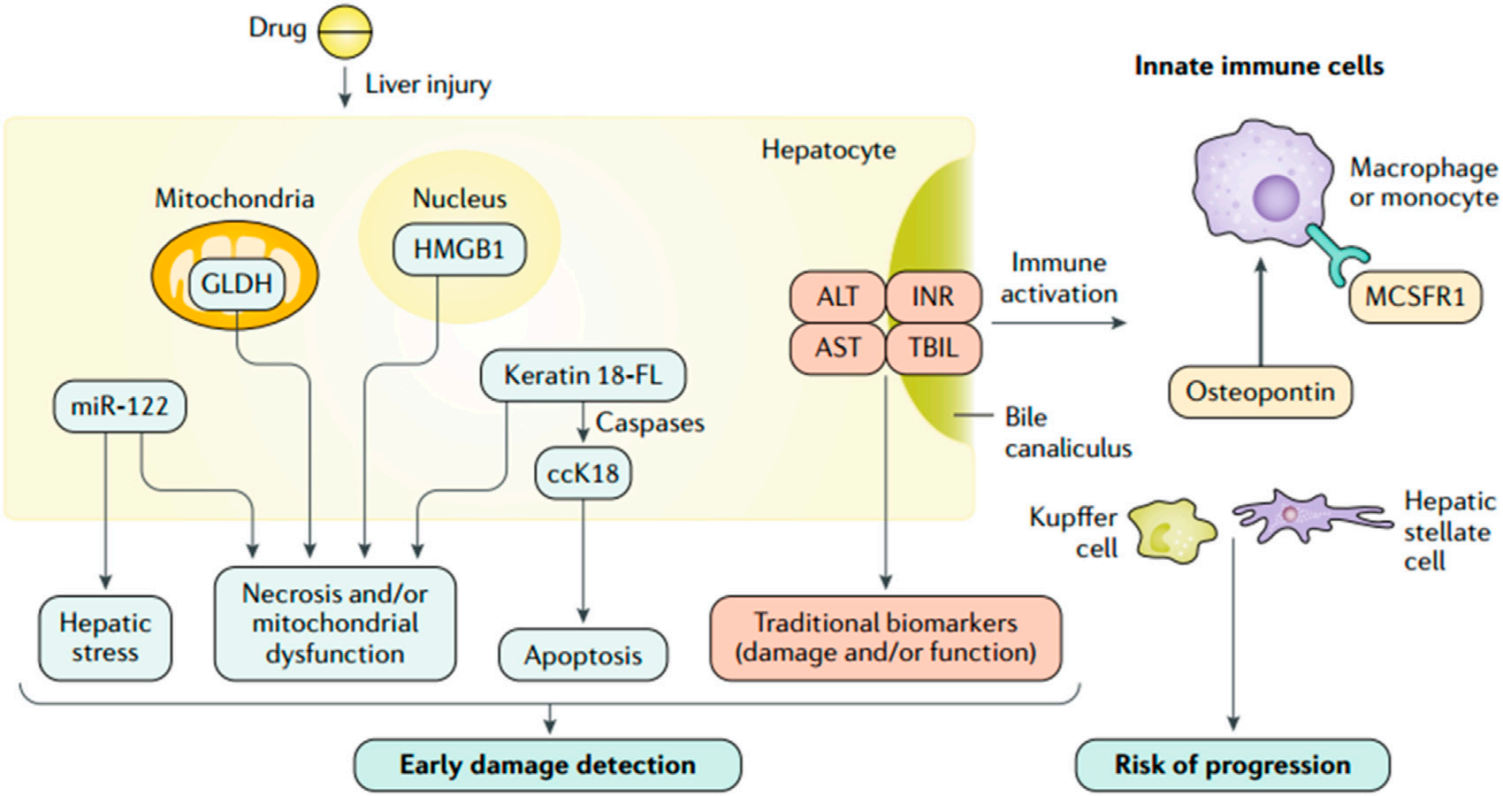
Traditional and investigational DILI biomarkers (Andrade et al., 2019a; reprinted from Nature Reviews Disease Primers with permission).

In the past 50 years, the main serum biomarkers used to screen and monitor DILI include ALT (Ozer et al., 2010), AST (Moreno-Torres et al., 2022), ALP (Watkins et al., 2008), and total bilirubin (TBIL). The degree of hepatocyte and biliary tract cell damage is clinically assessed based on the magnitude of their concentration elevation (Ravindra et al., 2023). Among them, ALT, as a marker of liver damage, was identified as the main parameter of DILI by the US FDA in 2009. AST has lower specificity and sensitivity than ALT (Moreno-Torres et al., 2022) and is generally used as a supplemental indicator for those with normal ALT. ALP can specifically indicate a cholestatic liver injury and severe DILI (Watkins et al., 2008). TBIL can reflect liver function more directly than ALT, AST, and ALP, an essential indicator in DILI’s staging and prognosis assessment. However, these serum biomarkers neither identify DILI specifically nor indicate possible DILI before the clinical occurrence of liver injury during medication treatment (Church and Watkins, 2017) (Figure 3). In recent years, an increasing number of studies have been conducted on metabolic enzymes, cellular proteins, and microRNAs, which have led to the discovery of novel biomarkers that can be used for the diagnosis, screening, and prevention of DILI (Hosack et al., 2023). Osteopontin (OPN), cytokeratin 18 (CK18) (Korver et al., 2021), and macrophage colony-stimulating factor receptor (MCSFR) have been reported as potential prognostic biomarkers for DILI (Matheis et al., 2011). Glutamate dehydrogenase (GLDH) and microRNA-122 (miR-122) have also been reported to be liver-specific and could be used as alternative markers of ALT (Church et al., 2019; Moreno-Torres et al., 2022).

When cell necrosis occurs, intracellular molecules are passively released, known as the damage-associated molecular pattern (DAMP). DAMPs related to hepatocyte injury include adenosine triphosphate (ATP), CK18, high mobility group box-1 protein (HMGB1) (Luo et al., 2022), mitochondrial DNA (mtDNA), and N-formyl peptide, which play an essential role in the process of AILI (Mihm, 2018). During AILI, damaged hepatocytes release DAMPs, some of which bind to pattern recognition receptors and trigger transcriptional activation of chemokine and cytokine genes, thereby driving a chemokine- and cytokine-based cascade response to initiate a systemic inflammatory response (Jaeschke and Ramachandran, 2020). Among them, the Toll-like receptor 9 (TLR9)-mediated signaling pathway is activated by circulating free DNA (cfDNA), which leads to an immune response with higher intensity and longer duration (Mihm, 2018). Therefore, cfDNA is a promising biomarker for diagnosing and monitoring AILI. The descriptions, advantages, detection methods, and sample sources of some biomarkers are summarized in Table 1. cfDNA, including neutrophil extracellular traps (Nets), mtDNA, and nuclear DNA, is elevated in patients who overdosed on APAP (Mcgill et al., 2012). Sun et al. (2023) observed significantly higher cfDNA levels in DILI patients than in healthy volunteers. In addition, a solid association between cfDNA concentration and AILI occurrence is confirmed in mice, which indicates that cfDNA has the potential to predict AILI. While its specificity is inferior to that of ALT, its sensitivity is higher. Thus, a combination of ALT and cfDNA levels might be ideal for predicting AILI. In conclusion, cfDNA can predict AILI in animal models but needs further confirmation in clinical trials.

**TABLE 1:** Descriptions, advantages, detection methods, and sample sources of biomarkers.

Name	Abbreviation	Descriptions	Advantages	Detection methods	Sample sources	Refs
Alanine aminotransferase	ALT	Released into the blood after hepatocyte damage	High liver specificity	-	Serum	Ozer et al. (2010)
Aspartate aminotransferase	AST		-	-	Serum	Moreno-Torres et al. (2022)
Alkaline phosphatase	ALP		Identify cholestatic liver injury	-	Serum	Watkins et al. (2008)
Total bilirubin	TBIL	Inadequate ability to metabolize bilirubin after liver injury	A more direct reflection of liver function	-	Serum	Ravindra et al. (2023)
Cytokeratin 18	CK18	Deficiency of CK18 in hepatocytes leads to liver lesions	Identify the mechanism of hepatocyte injury	ELISA	Serum	Korver et al. (2021)
Osteopontin	OPN	OPN has chemotactic effects on macrophages and neutrophils	Serum OPN levels correlate with prognosis	Immunoassay	Serum	Korver et al. (2021)
Macrophage colony-stimulating factor receptor	MCSFR	Shedding from activated macrophages during DILI	Promising prognostic biomarkers for death/transplantation	Immunoassay	EDTA-Plasma	Matheis et al. (2011)
Glutamate dehydrogenase	GLDH	Macromolecular proteins found in the mitochondrial matrix, enriched in the	High liver specificity and Strong correlation with ALT level	Activity Assay	Serum	Moreno-Torres et al. (2022); Church et al. (2019)
MicroRNA-122	miR-122	A liver-specific miRNA that can leak from damaged cells	High liver specificity	RT-qPCR	Serum	Moreno-Torres et al. (2022); Church et al. (2019)
High mobility group box-1 protein	HMGB1	Released as stress signals and mediators of inflammation	-	ELISA	Serum	Luo et al. (2022)
Circulating free DNA	cfDNA	Released by damaged cells	Nice AILI prediction potential	-	-	Sun et al. (2023)

In addition to cfDNA, some other biomarkers have been extensively studied. Kodihalli et al. identified some protein biomarkers capable of detecting DILI with high accuracy in a large multicenter case-control study. The protein biomarkers include cytoplasmic aconitate hydratase (ACO1), argininosuccinate synthase (ASS1), fumarylacetoacetase (FAH), fructose-1,6-bisphosphatase 1 (FBP1) and carbamoylphosphate synthase (CPS1) (Ravindra et al., 2023). They also found that FBP1 in combination with glutathione S-transferase A1 (GSTA1) or leukocyte cell-derived chemotaxin 2 (LECT2) might be able to differentiate between DILI and ALI caused by non-pharmaceutical factors, although further clinical validation is required. Kwan et al. (2023) found that patients with AILI had significantly higher CPS1 levels than patients with non-APAP-induced liver injury. Additionally, CPS1 levels were higher in patients with AILI who ended up with liver transplantation or death within 21 days of hospitalization than those who survived. Moreover, transplanted or deceased patients demonstrated higher CPS1 levels on day 3 than on day 1, but no significant changes in either ALT or AST levels. This study further confirms that serum CPS1 can be used as a potential biomarker to assess the clinical outcome of patients with AILI.

In the past decade, using biomarkers to predict, monitor and diagnose drug-induced diseases during drug treatment or clinical trials has gradually gained wide attention among professionals (Matheis et al., 2011). Currently, circulating APAP concentrations and ALT levels are the critical parameters used to diagnose and monitor AILI in clinical practice, provided that the exact timing of APAP intake is known (Chiew et al., 2020). However, ALT is not highly sensitive and has a lag that makes it difficult to promptly indicate the progression of liver injury (Antoine et al., 2013). Therefore, new biomarkers that can detect liver injury accurately and promptly are needed in clinical practice. Furthermore, identifying novel biomarkers that can screen drugs with hepatotoxicity or predict DILI’s prognoses are promising research areas (Vazquez and Mcgill, 2021). In the future, combining artificial intelligence (AI) or genomics with experimental approaches may help us identify novel biomarkers.

### 3.2 Predictive models for screening DILI

32% of post-approval withdrawals and 22% of clinical trial failures are caused by drug hepatotoxicity (Watkins, 2011), suggesting that preclinical studies of drugs cannot reliably assess the risk of hepatotoxicity of new medications. This suggests an unmet need for improved *in vitro* models for DILI risk prediction.

**FIGURE 4:**
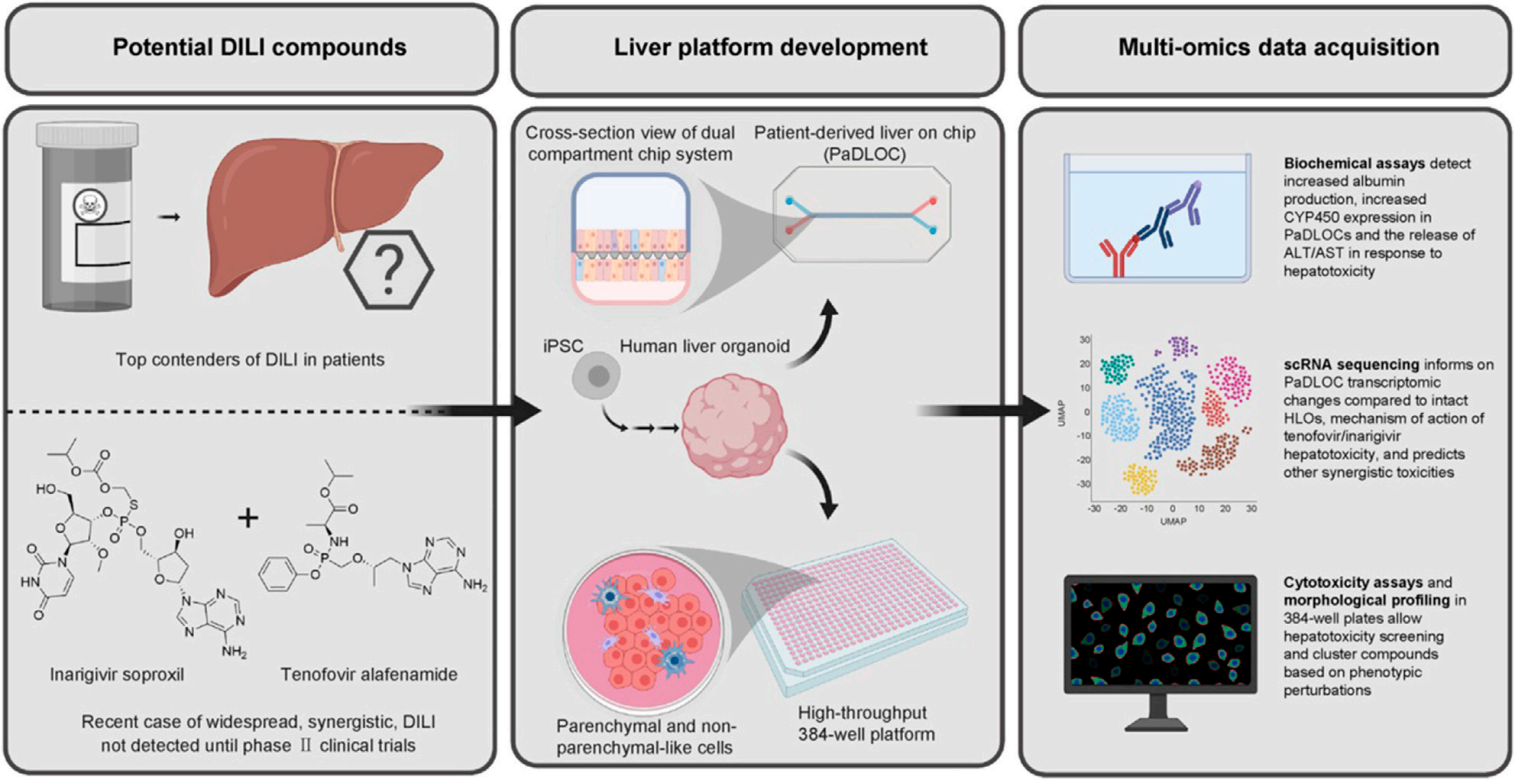
HLO-based screening platform for DILI risk prediction (Zhang et al., 2023; reprinted from Journal of Hepatology with permission).

Human liver organoids (HLOs) are cultured from adult stem/progenitor cells or pluripotent stem cells (PSCs), structurally and functionally similar to the human liver *in vivo*. HLOs are a more physiologically compatible organotypic system (Reza et al., 2021). Shinozawa et al. (2021) developed an HLO-based screening model to study the mechanism of DILI and susceptibility to individual drugs. They successfully developed an organoid-based model with high predictive values for 238 marketed drugs with cholestatic or mitochondrial toxicity. The model also succeeds in predicting genomic predisposition for certain types of DILI. Zhang et al. (2023) used HLOs to model *in vitro* liver function and identify hepatotoxic compounds in a 384-well plate-based high-throughput drug screening system (dispersed HLOs) and a liver-on-chip system (intact HLOs). They found dispersed HLOs had similar DILI predictive ability as intact HLOs during high-throughput screening. Significant morphological differences were observed when different types of hepatotoxic drugs were administered (Sheyn et al., 2019). Intact HLOs showed significantly increased albumin production, ALT/AST release, and CYP450 expression compared to dispersed HLOs when treated with hepatoxic drugs. Intact HLOs exhibited mitochondrial perturbation and steatosis when exposed to APAP (Agarwal et al., 2020). Therefore, HLOs demonstrated potential utility for DILI risk prediction. Figure 4 illustrates the steps to develop the HLO-based screening platform. In the future, HLO-based screening model accounting for diverse host genetics and other clinical factors can be developed based on this study (Takebe and Taniguchi, 2014).

### 3.3 Prevention of AILI

DILI has become a significant global health burden due to its high morbidity and mortality (Ostapowicz et al., 2002). NAC can effectively detoxify APAP in the early stages of liver injury by supplementing GSH. However, delayed administration of NAC may lead to failure of drug therapy, leaving patients with poor prognoses to eventually undergo liver transplantation (Heard, 2008). As drug treatment options for AILI are limited, great attention should be paid to the prevention of AILI.

The foundation of preventing DILI is vigilance during preclinical drug development and clinical trials. During drug development, AI-based DILI prediction models can help researchers initially screen for compounds that may be hepatotoxic. Deep learning (DL) architectures developed based on deep artificial neural networks have shown the ability to process big data with little human intervention and have been applied to the chemical and bioinformatics fields, which may be adopted in DILI predictions (Park and Kellis, 2015). Several studies have adopted AI methods in the past decade to establish DILI prediction models and demonstrate their capabilities and potential in predicting DILI (Xu et al., 2015). Chen et al. (2022) obtained a high-confidence DILI compound dataset after a comprehensive literature search and data screening, based on which a DILI prediction model founded on the residual network18 deep neural network (ResNet18DNN) was established (Tujios and Lee, 2018). The results showed that the area under the curve (AUC), accuracy, recall, F1 score, and specificity of the model were higher compared with published models in the last decade, indicating the superior performance of the ResNet18DNN model. The ResNet18DNN model is the best choice for predicting the hepatotoxicity of drugs and small molecule compounds. However, due to the individual variability of DILI, studies that consider genetic factors are needed to optimize the model subsequently. In addition, Shin et al. (2022) developed ToxSTAR, a web server for predicting DILI subtypes such as cirrhosis, hepatitis, cholestasis, and steatosis based on the structure of the drug and its metabolites *in vivo*. However, the accuracy of prediction results still needs further confirmation. Lim et al. (2023) proposed a novel DILI prediction method, supervised subgraph mining (SSM), which can identify compound subgraph populations and classify them according to the structural features to predict hepatotoxicity and mechanism of DILI. The results showed that the SSM method was highly accurate in predicting hepatotoxicity, but experimental validation is still needed.

During clinical trials, investigators must select patients carefully, thoroughly assess their liver function, closely monitor clinical symptoms and biochemical parameters, and establish clear rules for drug discontinuation. Furthermore, adopting specific methods to monitor ALT and other biomarkers is supposed to facilitate the prevention of DILI (S and N, 2009).

In addition, FDA’s publication Drug-Induced Liver Injury: Premarketing Clinical Evaluation provides investigators with laboratory results of drugs that could be referred to predict severe DILI during the drug development process. Suppose a drug identified as potentially hepatotoxic is still approved for marketing. In that case, it will be mandatory to indicate the safety information and treatment measures in the warnings, adverse reactions, and precautions sections. All of these requirements contribute to the prevention of DILI (Andrade et al., 2019a).

## 4 Management of AILI

**FIGURE 5:**
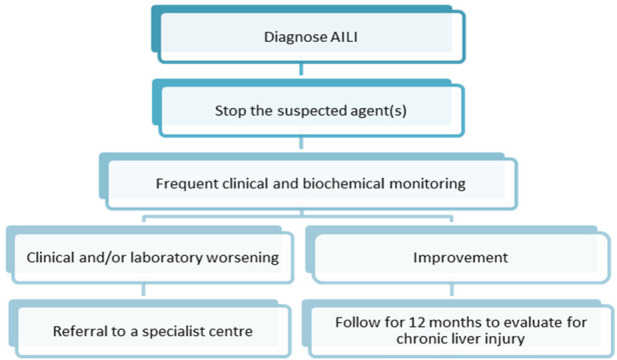
General management steps after the diagnosis of AILI.

The key to AILI management is the timely detection of liver injury, prompt discontinuation of offending medications, and referral of patients to advanced medical care in the early stage. Since AILI patients have the potential to develop chronic liver injury at a later stage, patients need to be followed up for at least 12 months after treatment. Biochemical parameters of liver function should be continuously monitored (Andrade et al., 2019a). Figure 5 describes the management steps after the diagnosis of AILI. Most AILI patients recover without additional aggressive treatment after discontinuing the offending medication. NAC is the primary option for patients with liver injury requiring pharmacological treatment (Heard, 2008).

**FIGURE 6:**
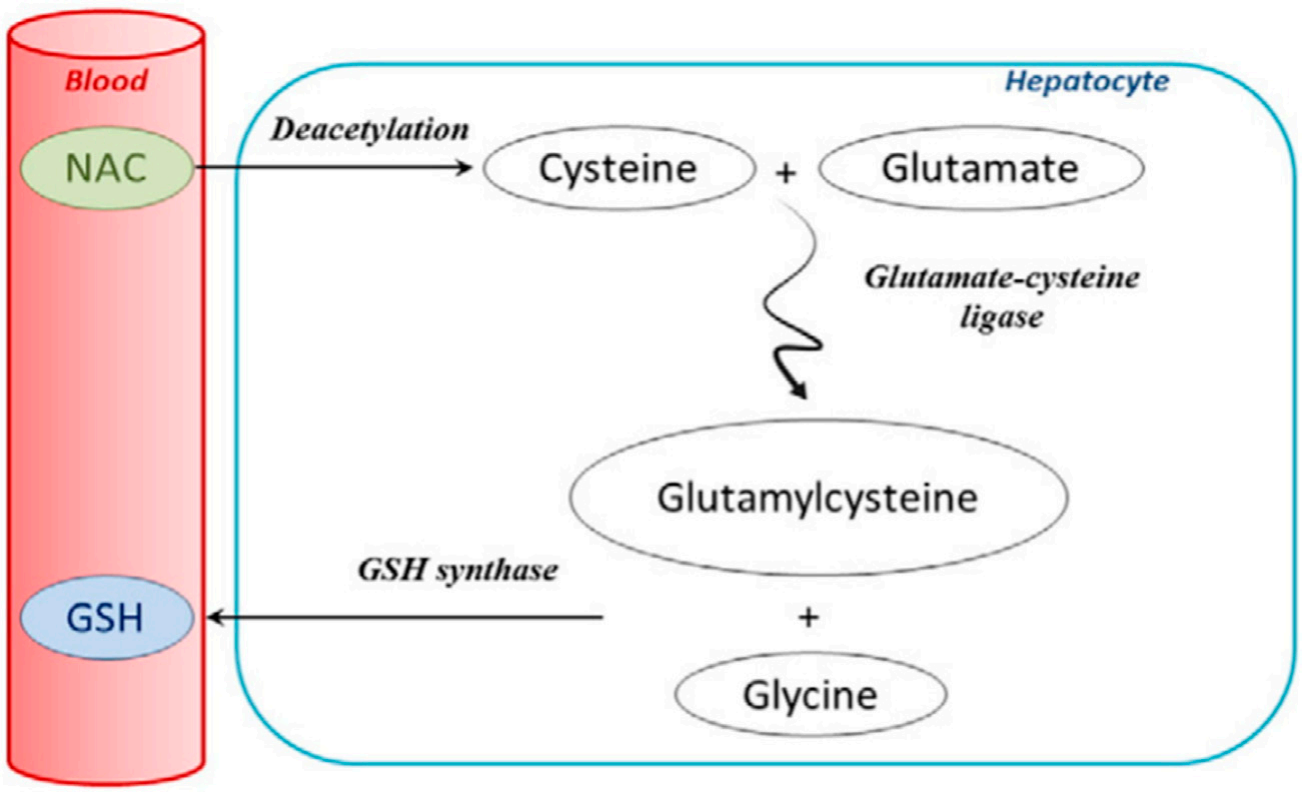
Extensive first-pass metabolism of NAC in the liver after oral administration (Lasram et al., 2015; reprinted from Clinical Biochemistry with permission).

Because of the absolute role of mitochondrial oxidative stress and dysfunction in the pathogenesis of AILI, the FDA approved the antioxidant NAC in 2004 to treat intrinsic DILI caused by excessive APAP intake. NAC is currently the only FDA-approved APAP antidote (Chowdhury et al., 2020). NAC is a precursor drug of GSH, which is firstly converted to cysteine by deacetylation when entering the body, and then further converted to glutamylcysteine by binding to glutamate in the action of glutamylcysteine synthase in hepatocytes. After that, glutamylcysteine is combined with glycine by the activity of GSH synthase and finally converted to GSH (Lasram et al., 2015) (Figure 6). NAC supplements the amount of intracellular GSH to reduce the covalent binding of NAPQI to cellular proteins, thus reducing hepatocyte necrosis. In addition, it has been shown that NAC can also reduce the inflammatory response in the liver and improve mitochondrial energy metabolism by maintaining the amount of ATP (Saito et al., 2010; de Andrade et al., 2015). However, the use of NAC has some limitations. First, NAC is ineffective if patients overdose on APAP but seek medical help too late when excessive liver injuries have already occurred. The patient may need to receive liver transplantation instead (Craig et al., 2010). Secondly, adverse effects, including headache, tinnitus, and urticaria, may be caused by NAC. This suggests an unmet need for treatment options of AILI superior to NAC in clinical practice.

Nanoparticle-mediated drug delivery systems have promising applications in treating liver diseases by coupling specific ligands that bind to receptors on the surface of hepatocytes, reducing drug hepatobiliary clearance (Kang et al., 2016; Zhang et al., 2016; D'Souza and Devarajan, 2015). The asialoglycoprotein receptor (ASGPR) is one of the specific receptors on the surface of hepatocytes. It has a high affinity for galactose, lactose, and glucose (Bon et al., 2017). Porterfield et al. (2023) synthesized a polymer named D4-Gal, which binds to ASGPR and selectively targets hepatocytes in healthy mice and AILI mouse models. They further synthesized Gal-D-NAC, a D4-Gal conjugate containing NAC, and tested it in the above model. The results showed that Gal-D-NAC could reduce cellular oxidative stress and shrink hepatocyte necrotic areas, thus improving the survival rate of AILI mice. Specifically, the effectiveness persisted when Gal-D-NAC was administered 8 h after excessive APAP exposure (Porterfield et al., 2023).

Natural products are effective in treating AILI, often exerting therapeutic effects through attenuating cellular damage caused by oxidative stress, activating the Nrf2 signaling pathway, reducing the release of inflammatory factors, and regulating GSH synthesis, coupling, and excretion (Sun et al., 2022). Astaxanthin (ASX) is a ketocarotenoid with more potent antioxidant activity than other carotenoids (Ambati et al., 2014). Natural ASX has been reported to significantly reduce oxidative stress and clear free radicals, thus reducing inflammatory responses and DNA damage (Kumar et al., 2022). Cai et al. (2022a) explored the interaction between the biological activity of ASX and the mechanism of AILI. The results showed that ASX effectively ameliorated AILI by attacking the NF-κB pathway to reduce inflammation, inhibiting oxidative stress and iron death, and activating the Nrf2/HO-1 pathway to increase mitochondrial autophagy. Moreover, urolithin is a natural metabolite obtained from the intestinal microbiota’s catabolism of ellagitannin (ET) and ellagic acid (EA). Among the metabolites of ET, urolithin A (UA) exhibits the most significant biological activity and hepatoprotective effects (Espín et al., 2013). Gao et al. (2022) investigated the therapeutic effects and potential molecular mechanisms of UA on AILI on this basis. The results indicated that UA attenuated APAP hepatotoxicity by suppressing excessive APAP-induced cellular oxidative stress through sustained activation of the Nrf2/ARE signaling pathway. In addition, UA has a lower therapeutic dose and a broader therapeutic window than NAC.

However, something needs to be considered for the use of natural products. Many of these products are required to be administered in high or repeated doses to achieve the desired therapeutic effect and also need to be administered in combination with high concentrations of DMSO. However, this may lead to an increased incidence of adverse reactions and may also lead to off-target or toxic reactions, decreasing patient compliance, which is an issue that needs to be addressed. In addition, because the mechanism of some natural products is to induce Nrf2 before APAP administration, they need to be given in advance. Therefore, natural products can be used prophylactically to protect the liver from serious liver injury in patients highly susceptible to hepatotoxic drugs. Natural products are a treasure trove of drugs worth developing and utilizing. In the future, investigating new drugs using natural products as the source will be one of the critical approaches for AILI treatment (Zhou et al., 2021). The various components of natural medicines provide multiple targets for disease treatment. Therefore, the rational use of independent or synergistic effects of natural drugs could offer more approaches for AILI treatment, which needs further investigation by clinical trials.

## 5 Summary and outlook

First, the review describes the common classification of DILI, the clinical manifestations of different types of liver injury, and the current research on biochemical indicators. Then research progress in the pathological mechanisms of AILI is discussed. It is now recognized that the pathological process of AILI includes multiple intra- and extracellular activities, such as mitochondrial oxidative stress, endoplasmic reticulum stress, aseptic inflammation, bile acid cycle, and microcirculatory dysfunction (Cai et al., 2022b). Therefore, many processes other than mitochondrial oxidative stress and dysfunction can be potential targets for AILI treatment. The review also summarizes several signaling pathways investigated in recent years, such as the SRA-IL-10 axis and HNF4α-cMyc, to provide more options for AILI treatment. The study focuses on an overview of currently adopted measures for diagnosis, screening, prevention, and management of AILI. It summarizes new findings on biomarkers, *in vitro* predictive models, and prevention and treatment of AILI. Biomarkers commonly used to screen and monitor for DILI are ALT, AST, ALP, and TBIL, but they are not DILI-specific and cannot predict liver injury. Some novel biomarkers, such as cfDNA and CPS1, are discussed, which can predict liver injury accurately and promptly. Hepatotoxicity is often one of the main reasons for drug discontinuation and treatment failure. *In vitro* prediction models indicate liver injury risks and facilitate identifying hepatotoxic compounds during drug development process. HLO-based screening platforms are discussed as an example. Currently, there are few and limited medication treatments for APAP overdose. Although NAC exerts a detoxification effect in the early stage of liver injury, delayed administration may lead to the failure of pharmacotherapy. Therefore, prophylactic and therapeutic approaches for AILI are still in need. The review indicates the prospect of nanoparticle-mediated drug delivery systems in AILI treatment, such as Gal-D-NAC. Natural products are a rich reservoir of drugs, and the study introduces ASX and UA to provide more options for new drug development.

Despite the tremendous progress made in studying AILI, there are still barriers to fully understanding and effectively managing AILI due to the complicated mechanisms involved in APAP hepatotoxicity. Firstly, the accurate diagnosis of DILI is an essential prerequisite in clinical treatment. However, diagnosing DILI is challenging due to the lack of diagnostic biomarkers that can identify DILI specifically. In clinical practice, DILI remains a diagnosis based on excluding other possible causes. In addition to assessment methods such as the Roussel Uclaf Causality Assessment Method (RUCAM) (Andrade et al., 2019b), the RECAM, an updated scoring system for computer-aided diagnosis (not yet validated) (Huo et al., 2017; Hayashi et al., 2022), the Clinical Diagnostic Scale (CDS) (Maria and Victorino, 1997) and the Structured Expert Opinion Process (not yet externally validated) (Rockey et al., 2010), an unmet need exists for advances in diagnostic methods. Secondly, although many *in vitro* prediction models with high accuracy are available, generalizing the characteristics of DILI in a single *in vitro* model and using it for screening and disease prediction is difficult due to the individual variability of DILI (Weaver et al., 2020). This is because predictive models must be developed based on the DILI mechanism, be sufficiently adaptable to various situations, and predict risks early in the drug development process. However, the currently available models can only meet some of these requirements. Finally, although natural products are effective in treating AILI, reducing the toxic effects of natural products is challenging. Moreover, a high or repeated administration dose is required to achieve the desired therapeutic effect. In that case, it may lead to off-target or toxic reactions, decreasing patient compliance (Traverso and Langer, 2015). In addition, the need for natural product monomers to cross multiple biological barriers and resist degradation by various enzymes limits their efficacy (Homayun et al., 2019).

AI research, which takes advantage of big data and machine learning, is a relatively new method for diagnosing and predicting DILI, which can help identify new drugs under development with hepatotoxicity to reduce drug development costs in the early stage (Vall et al., 2021). Another example would be quantitative systems pharmacology (QSP), which can predict DILI risks based on liver physiology, referring to toxicity data obtained from *in vitro* experiments (Woodhead et al., 2017). In addition, histology-based high-throughput technologies improve the efficiency of identifying novel biomarkers (Youhao et al., 2022). These topics could be directions for further research. A large predictive DILI platform can be subsequently founded to integrate established and currently emerging models. Finally, hydrogels with good biocompatibility, low immunogenicity, and modifiable physical and chemical properties can be ideal carriers to improve efficacy and reduce the toxicity of natural products (Nicodemus and Bryant, 2008; Yegappan et al., 2018; Pushpamalar et al., 2021).

In conclusion, DILI cannot be eliminated as an adverse drug reaction, and our ultimate goal is to minimize its impact on clinical outcomes, and patient health. This review is supposed to serve as a reference and guidance for diagnosing, screening, preventing, and managing AILI in clinical practice. Moreover, providing directions and potential methods for pharmaceutical companies to develop novel drugs is expected.
