# Corrigendum: Advances in the study of acetaminophen-induced liver injury

**DOI:** 10.3389/fphar.2023.1283596

**Published:** 2023-09-21

**Authors:** Xinghui Li, Jiaqi Ni, Li Chen

**Affiliations:** ^1^ West China School of Pharmacy, Sichuan University, Chengdu, China; ^2^ Department of Pharmacy, Evidence-Based Pharmacy Center, West China Second University Hospital, Sichuan University, Chengdu, China; ^3^ Key Laboratory of Birth Defects and Related Diseases of Women and Children, Sichuan University, Ministry of Education, Chengdu, China

**Keywords:** drug-induced liver injury, acetaminophen-induced liver injury, diagnosis, screening, prevention and management

In the published article, there was an error in the caption for Figures 2, 3, 4, 6 as published. The previous captions for these did not attribute the figures to their citing source. The corrected figure captions appear below.

**FIGURE 2:**
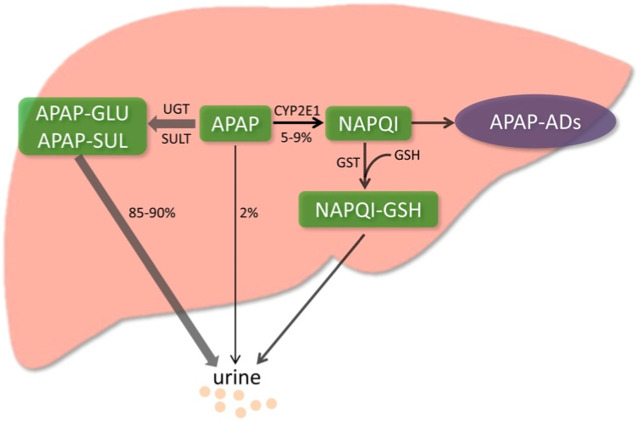
Metabolic activation pathway of acetaminophen. Generally, NAPQI is detoxified by conjugating with GSH. However, excessive NAPQI depletes GSH following APAP overdose, leading to the formation of APAP protein adducts (APAP-Ads) through the covalent binding of sulfhydryl groups in cellular proteins. (**Yan et al., 2018**; reprinted from Redox Biology with permission).

**FIGURE 3:**
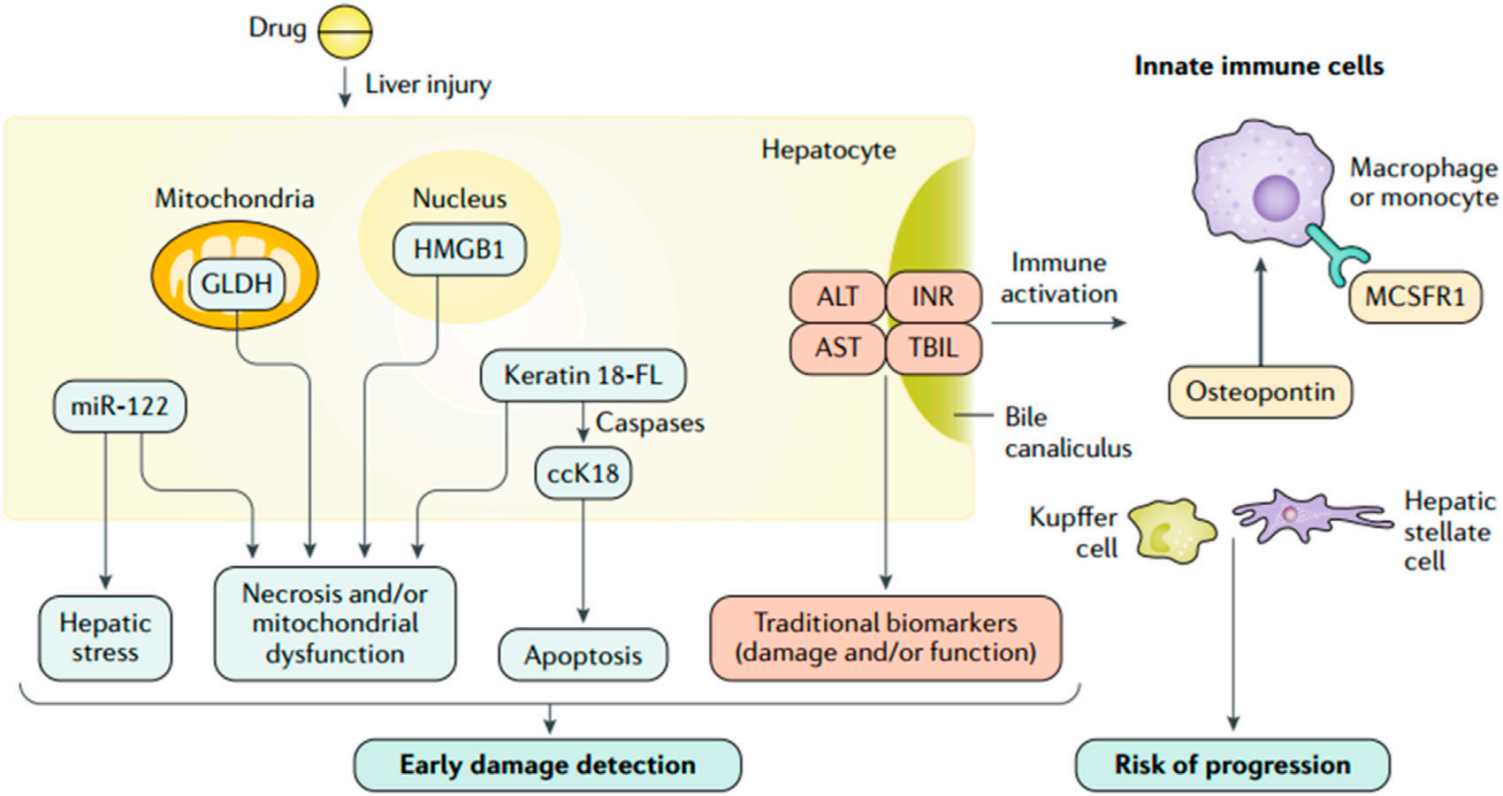
Traditional and investigational DILI biomarkers (**Andrade et al., 2019a**; reprinted from Nature Reviews Disease Primers with permission).

**FIGURE 4:**
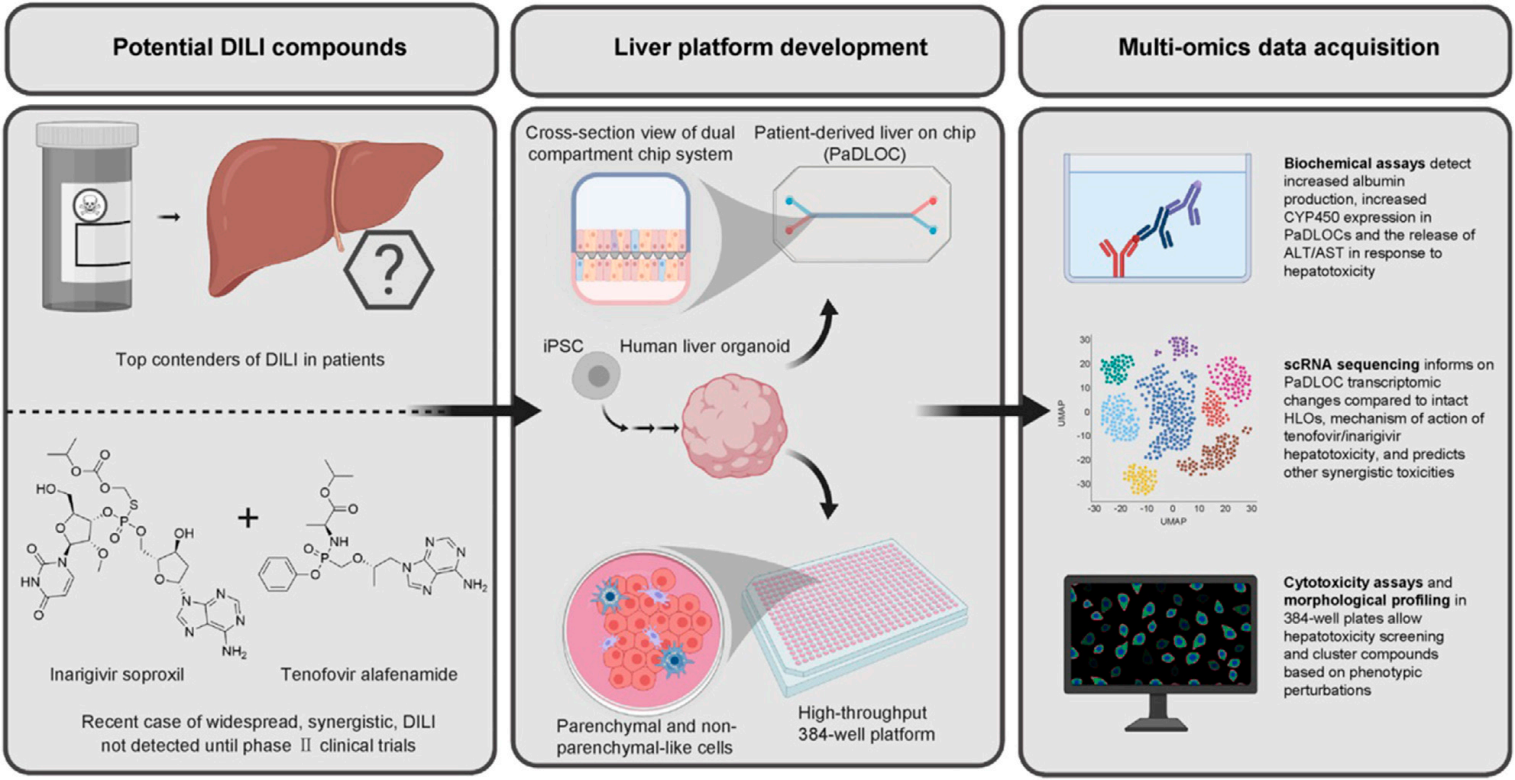
HLO-based screening platform for DILI risk prediction (**Zhang et al., 2023**; reprinted from Journal of Hepatology with permission).

**FIGURE 6:**
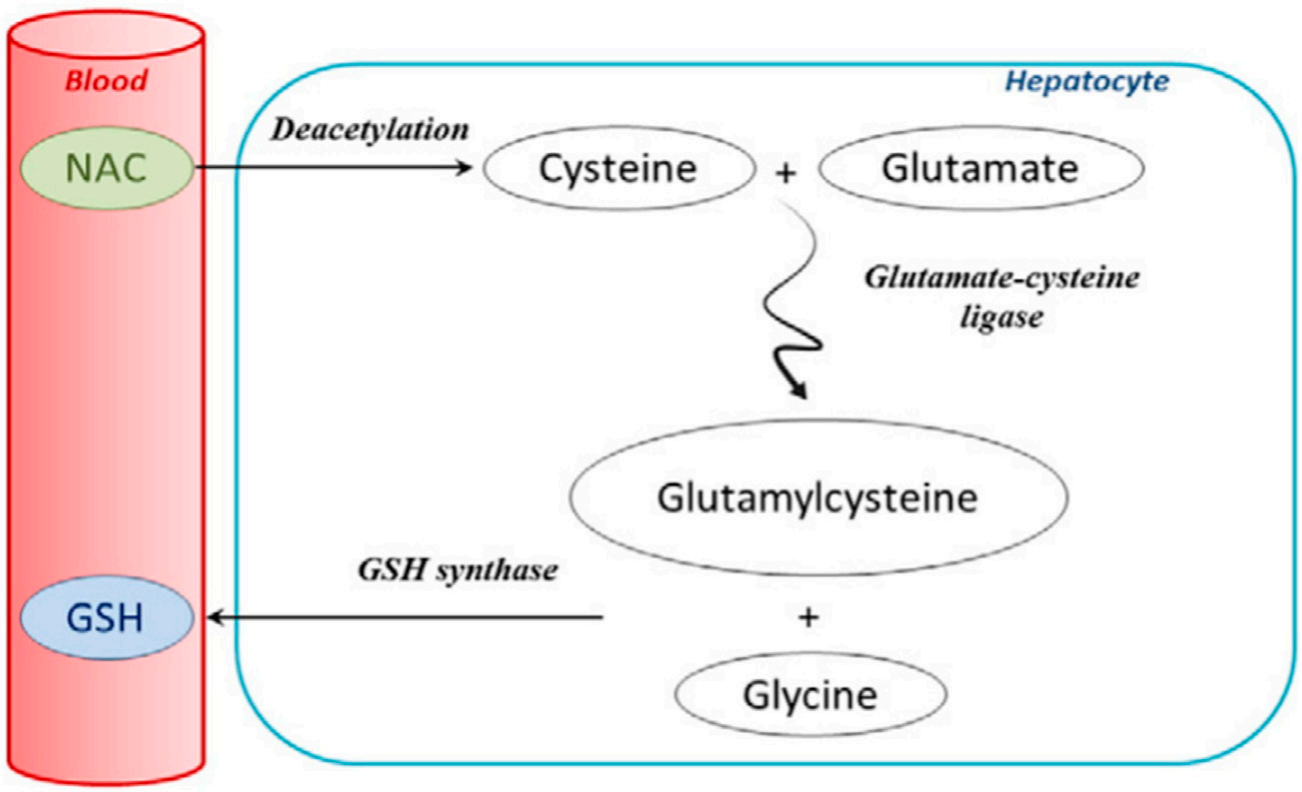
Extensive first-pass metabolism of NAC in the liver after oral administration (**Lasram et al., 2015**; reprinted from Clinical Biochemistry with permission).

The authors apologize for this error and state that this does not change the scientific conclusions of the article in any way. The original article has been updated.

